# HTLV-1 proviral load of HAM/TSP patients according to new diagnostic criteria of HAM/TSP

**DOI:** 10.1186/1742-4690-8-S1-A248

**Published:** 2011-06-06

**Authors:** Maria F R Grassi, Viviana N Olavarria, Ramon A Kruschewsky, Rita Elizabeth Mascarenhas, Inês Dourado, Luis C L Correia, Carlos M de Castro-Costa, Bernardo Galvão-Castro

**Affiliations:** 1 Advanced Laboratory of Public Health, Fundação Oswaldo Cruz /Bahia, Salvador, Bahia, Brazil; 2 Escola Bahiana de Medicina e Saúde Pública, Bahia, Brazil; 3 Instituto de Saúde Coletiva, Federal University of Bahia, Bahia, Brazil; 4 In memoriam, Laboratory of Experimental Neurology and Neurophysiology, Federal University of Ceará, Fortaleza, Ceará, Brazil

## Introduction

A high HTLV-1 proviral load is described in HTLV-1-associated diseases, especially HAM/TSP. However, the cutoff value to define high levels of HTLV-1-proviral load is not well established.

## Methods

281 HTLV-1-infected patients from the HTLV reference center in Salvador, Brazil, were followed from 2005 to 2008. Patients were classified as asymptomatic, possible-, probable- and definite-HAM/TSP, in accordance with diagnostic criteria proposed by De Castro-Costa et al 2006. HTLV-1- proviral load was determined using real-time PCR. A receiver operator characteristic (ROC) curve was constructed using only asymptomatic individuals and definite-HAM/TSP patients. The ROC curve was used to predict the proviral load level that differentiates these two groups.

## Results

Out of 281 patients, 189 were asymptomatic and 92 were diagnosed with HAM/TSP (22 possible, 23 probable, 47 definite). The mean HTLV-1 proviral load was higher in possible-(89,104±93,006 copies/10^6^ PBMC), -probable (175,854±128,083 copies/10^6^ PBMC) and definite- HAM/TSP patients (150,667±122,320 copies/10^6^ PBMC), when compared to asymptomatic individuals (27,178±41,155 copies/10^6^ PBMC) (p < 0.0001). A comparison of all HAM/TSP groups showed the highest proviral loads in probable-HAM/TSP patients, yet the differences in mean values were not statistically significant. The ROC curve suggested a value of 49,865 copies/ 10^6^ PBMC, with 87% sensitivity (95% CI= 74 to 95) and 81% specificity (95% CI= 75 to 86), as the HAM/TSP patients (150,667±122,320 copies/106 14 PBMC), when compared to asymptomatic individuals (27,178±41,155 copies/106 15 PBMC) (p < 0.0001). A comparison of all HAM/TSP groups showed the highest proviral loads in probable-HAM/TSP patients, yet the differences in mean values were not statistically significant. The ROC curve suggested a value of 49,865 copies/10^6^ PBMC, with 87% sensitivity (95% CI= 74 to 95) and 81% specificity (95% CI= 75 to 86), as the best proviral load cutoff point to differentiate definite HAM/TSP patients from asymptomatic individuals.

